# Organoid models: applications and research advances in colorectal cancer

**DOI:** 10.3389/fonc.2025.1432506

**Published:** 2025-02-07

**Authors:** Yijie Wu, Yu Sha, Xingpo Guo, Ling Gao, Jian Huang, Song-Bai Liu

**Affiliations:** ^1^ College of Life Science, North China University of Science and Technology, Tangshan, China; ^2^ Jiangsu Province Engineering Research Center of Molecular Target Therapy and Companion Diagnostics in Oncology, Suzhou Vocational Health College, Suzhou, China; ^3^ Department of General Surgery, The First Affiliated Hospital of Soochow University, Suzhou, China

**Keywords:** organoids, colorectal cancer, research advances, cancer, applications

## Abstract

This review summarizes the applications and research progress of organoid models in colorectal cancer research. First, the high incidence and mortality rates of colorectal cancer are introduced, emphasizing the importance of organoids as a research model. Second, this review provides a detailed introduction to the concept, biological properties, and applications of organoids, including their strengths in mimicking the structural and functional aspects of organs. This article further analyzes the applications of adult stem cell-derived and pluripotent stem cell-derived organoids in colorectal cancer research and discusses advancements in organoids for basic research, drug research and development, personalized treatment evaluation and prediction, and regenerative medicine. Finally, this review summarizes the prospects for applying organoid technology in colorectal cancer research, emphasizing its significant value in improving patient survival rates. In conclusion, this review systematically explains the applications of organoids in colorectal cancer research, highlighting their tremendous potential and promising prospects in basic research, drug research and development, personalized treatment evaluation and prediction, and regenerative medicine.

## Introduction

Colorectal cancer (CRC) (1) is one of the most severe diseases and is a secondary cause of cancer-related death worldwide (2). Fifty-six percent of patients with CRC die from this disease (3), and for those with advanced metastatic colorectal cancer, the survival rate is only 4% to 12% (4). Compared with healthy colorectal cells, colorectal cancer cells undergo significant and frequent somatic mutations, and the genetic diversity of the cancer leads to prominent biological heterogeneity (5). Although the current preclinical study model, which includes cell lines, animal models, and clinical samples, has been used to contribute to the understanding of CRC, it still has certain limitations (6). To address these limitations, the organoid model provides a more advanced scientific tool (7).

The organoid model is an *in vitro* cultured three-dimensional tissue structure that mimics the microarchitecture of organs *in vivo*. Models can be derived from primary tissue samples, embryonic stem cells, somatic stem cells, or pluripotent stem cells (8, 9). Organoids contain many organ-specific cell types and exhibit spatial organization arrangements similar to those of their *in vivo* counterparts. This structural property endows organoids with a significant advantage in mimicking organ function. Organoids are considered advanced *in vitro* cancer models that can efficiently recapitulate the tumor microenvironment and maintain the heterogeneity of cell populations (10, 11).

In 2009, Sato, T, and colleagues first confirmed that single Lgr5+ intestinal stem cells (ASCs) are capable of self-organizing and differentiating to form crypt–villus structures that encompass all intestinal cell types (12). This discovery effectively replaced traditional cell lines and animal models; although this study has led to a breakthrough in stem cell research, the understanding of stem cells is still limited, and more research is needed to gain a deeper understanding of their differentiation mechanisms and regulatory networks (13, 14). As effective models for colorectal cancer research, organoids exhibit complex three-dimensional structures, cell heterogeneity, self-renewal, and self-organization (15). The transplanted organ plays a protective role by regulating the self-renewal of intestinal stem cells and modulating the immune microenvironment in recipient mice. For example, Fang-Ling Zhang’s research team reported that intestinal organ transplantation, as a therapeutic strategy, attenuates intestinal I/R injury in mice by promoting the self-renewal of intestinal stem cells and modulating the immune microenvironment. L-Malic acid (MA)-mediated polarization of M2 macrophages is dependent on SOCS2, a finding that provides a new understanding for the treatment of intestinal I/R injury (16).

To date, organoid models featuring diverse pathological characteristics, such as genetic diseases (17, 18), host− interactions (18), cancer (19, 20), intestinal hyperplastic polyps (21, 22) and gastrointestinal metaplasia (23), have been successfully developed, which further highlights the effectiveness of organoids in reflecting the biological characteristics of the colorectum and aids in deepening our understanding of the mechanisms underlying colorectal disease development. This also validates the methods used to develop these models, which are fundamentally capable of demonstrating high fidelity and genetic consistency (24).

## Types of organoids

### The biological properties of organoids derived from adult stem cells

Adult stem cells (ASCs) are a class of cells that possess the capacity for self-renewal and multidirectional differentiation, with the primary function of facilitating tissue repair and regeneration within the body (45). These cells are capable of differentiating into a variety of cell types. These compounds have demonstrated significant therapeutic effects in clinical treatment. It has become the gold standard in the field of stem cell research and therapy because of its minimal ethical controversy, broad acceptance, and successful application in treating patients.

Organoids derived from ASCs can regenerate *in vitro*, and they are primarily responsible for maintaining and repairing tissue functions (46, 47), which are closely associated with the development of colorectal cancer and have mutations and aberrant differentiation that may lead to tumor formation. According to current scientific research, colorectal organoids are capable of replicating the physiological state or degenerative conditions of the original tissue (48). FGFBP1 is overexpressed in colon, pancreas, breast, and skin cancers, and its expression accelerates skin wound healing in mice with conditional overexpression. Therefore, Fgfbp1 functions as a soluble autocrine/paracrine factor that mediates cross-talk between the epithelial stem/progenitor compartment and the niche, thereby reinforcing Fgfbp1+ cell identity and self-renewal (49). For example, Claudia Capdevila’s research team has recently achieved significant progress in the use of intestinal epithelial organoids. These findings indicate that FGFBP1-labeled pluripotent stem cells are capable of generating Lgr5+ cells, which can maintain the regenerative capacity of the intestinal epithelium when Lgr5+ cells are depleted (5). This research has challenged the traditional understanding of intestinal stem cells and revealed the important role of Fgfbp1+ cells in intestinal epithelial regeneration.

In a previous study, Johanna F Dekkers and colleagues employed biopsied intestinal organoids to investigate a disease resulting from a mutation in CFTR (50). However, organoid models at that time could not fully replicate the intricacies of cancer development, including tissue structure, cellular diversity and *in vivo* homeostasis. Consequently, the study of tumorigenesis *in vitro* is challenging, and the use of alternative models would incur significant costs. With the advent of organoids, many studies have been conducted to address this gap. Most recently, L. F. Lorenzo-Martín’s research team developed a topologically and biologically complex mini-colon organoid. It can be guided to cancer by blue light irradiation-activated spatiotemporally controlled tumorigenic transformation and can track neoplastic colon tumors in real time for weeks at single-cell resolution without destroying the organoid. The induced mini-colonic organoids demonstrate rich intratumoral and intertumoral diversity, as well as the physiological features characteristic of key pathologies of colorectal tumors *in vivo (* 51). This study effectively bridges the gap between tumor formation *in vitro* and provides a novel approach to cancer initiation, colorectal cancer lesions, and mechanisms of development in living organisms *in vitro (* 52).

The initial materials for isolating adult and embryonic stem cells were patient-derived tumor tissues and mouse models. The final stage of the process results in the formation of organoids, which represent the primary end product of this research. Organoids are prominent in several application areas, including but not limited to, basic biological research, drug toxicity assessment, the construction of disease models, the creation of organoid biobanks, and the development of organoid microarray technology, which are also expected to have positive impacts on human health and the advancement of science. Organoid research is being used to mimic human disease states, which will provide an in-depth understanding of disease mechanisms.

### The functional properties of organoids derived from pluripotent stem cells

**Figure 1:**
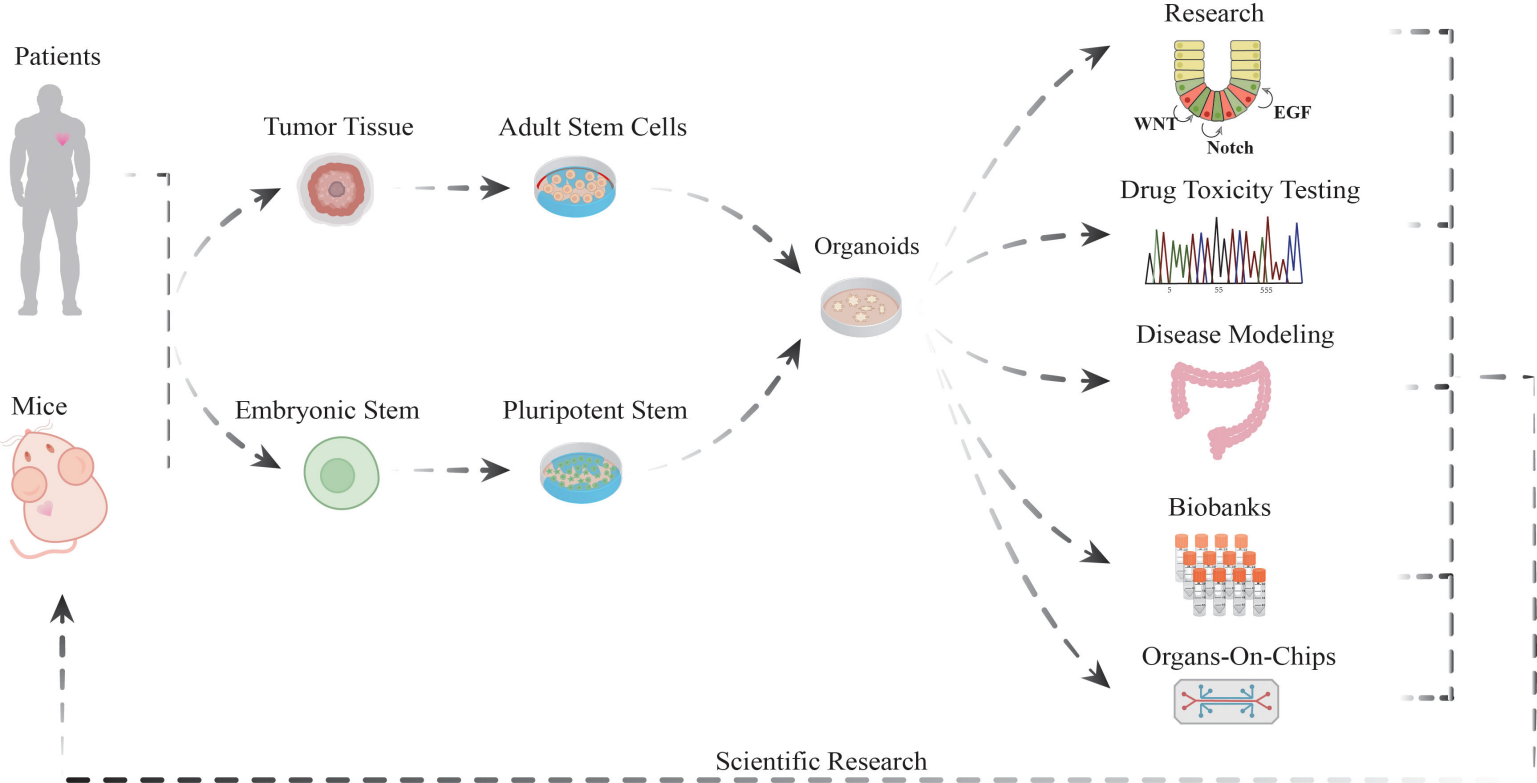
The versatile applications of organoid models in biomedical research.

It is widely acknowledged that pluripotent stem cells (**Figure 1**) are capable of differentiating into cell types derived from the three embryonic germ layers (53, 54). These cells possess significant developmental potential and are acquired through both embryonic stem cells (ESCs) and induced pluripotent stem cells obtained through reprogramming (55) (**Figure 1**). The reprogramming of somatic cells to pluripotent stem cells can be achieved through two main strategies: the transduction of specific combinations of transcription factors [e.g., SOX2 (56)] and the application of small molecule compounds to induce pluripotency. The potential applications and research value of pluripotent stem cell technology are extensive, spanning the fields of pathophysiology modeling, drug research and development, and regenerative medicine (57).

Researchers have successfully differentiated human colonic organoids from pluripotent stem cells, which closely resemble the human colon in both structure and function. This provides a powerful *in vitro* model for the study of colorectal diseases (58). For example, Xiaobo Zheng’s research team discovered that the overexpression of BMX and HCK markedly enhanced the proliferation of colorectal epithelial cells. Further studies demonstrated that the upregulation of BMX and HCK activated the JAK-STAT signaling pathway, resulting in the formation of multilayered polypoid structures that mimic the pathologic polyps commonly found in colorectal cancers (59). These findings provide a theoretical foundation for the early prevention and development of new therapies for CRC epithelial cell transformation. In the field of human colorectal organoid research, pluripotent stem cells can be differentiated into cell types with specific functions through the guidance of specific factors (60). For example, the results of Pilar Bustamante-Madrid and colleagues suggested that the BMP and Notch signaling pathways play pivotal roles in directing the differentiation of human colonic stem cells toward enterocyte and goblet cells. Furthermore, the modulation of these processes by calcitriol has also been demonstrated to contribute to the maintenance of stemness traits (61). Organoids contribute to understanding the role of factors and signals in the human colonic epithelium, which is relevant to the study of intestinal pathologies, including colon cancer and inflammatory bowel disease (ulcerative colitis and Crohn’s disease). Organoids can mimic not only the process of human intestinal development but also key cell types and structural features present during intestinal development. For example, Na Qu and colleagues constructed human colonic organoids (HCOs) and human intestinal organoids (HIOs) via human pluripotent stem cells. The removal of BMP signaling or the addition of the inhibitor NOGGIN facilitated the formation of organs resembling the developing small intestine and colon, as well as primary epithelial and mesenchymal cells within the colon (62). These results elucidate the mechanisms of intestinal regional specification and provide a powerful tool for studying intestinal development, disease modeling, and drug screening.

## The versatile applications of organoids

### Basic research: the applications of organoids in CRC

**Figure 2:**
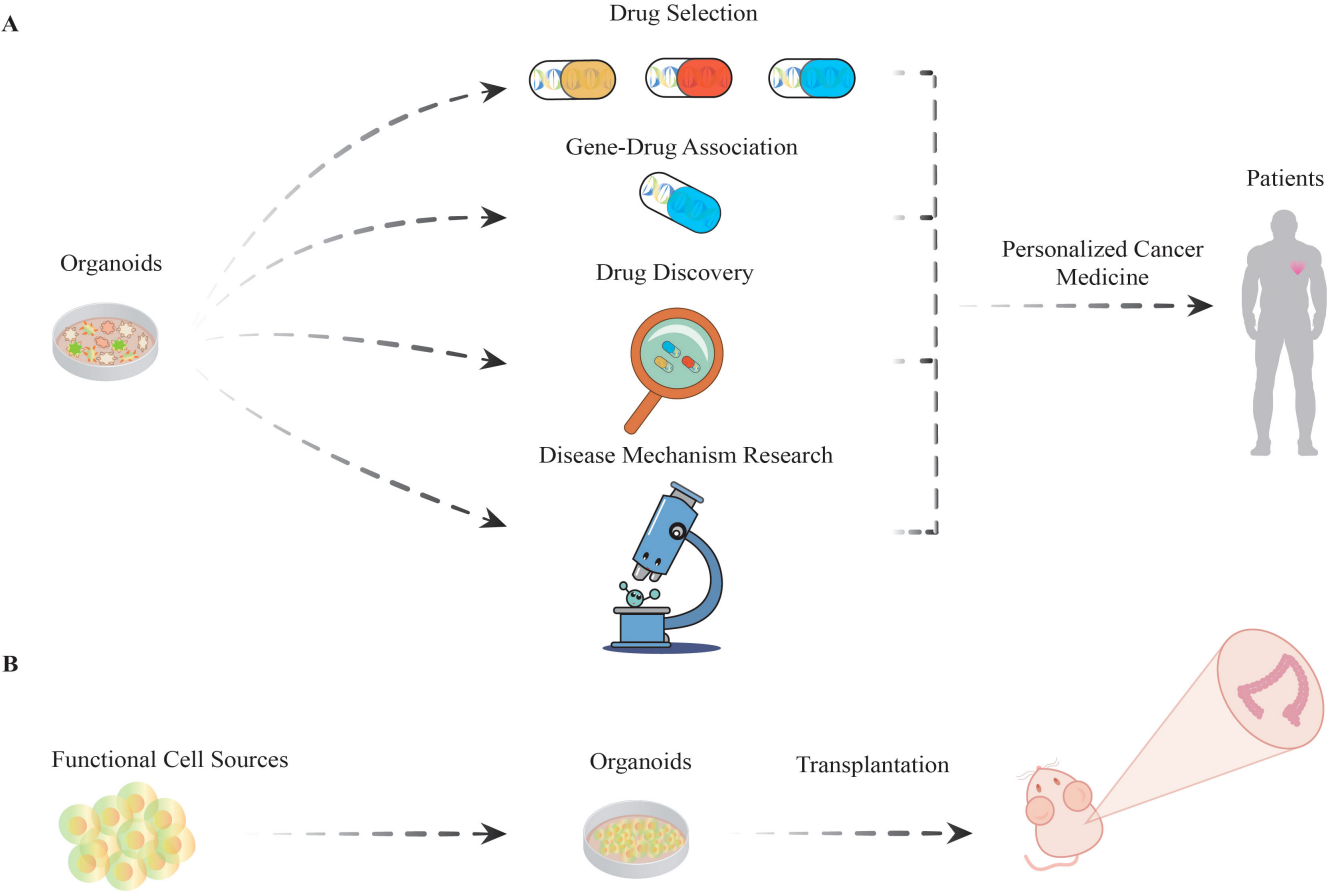
The versatile applications of organoids. **(A)** Organoids are employed to identify drugs with optimal efficacy among various drug candidates. Subsequently, drug−gene interactions are investigated to elucidate the correlation between specific gene mutations and drug responsiveness. On this basis, research will progress to the drug development phase, with in-depth analyses of disease mechanisms. Ultimately, the integration of cumulative research results will be used to construct personalized treatment protocols aimed at improving therapeutic efficacy and reducing the incidence of adverse effects, thereby increasing patient survival. **(B)** Organoids have the potential to be utilized in regenerative medicine as functional cell sources. This involves directing stem cells with multidirectional differentiation potential to develop into organoids, which are subsequently transplanted into mouse models to replicate and study functional and differentiation processes in living organisms.

By 2024, the number of articles related to organoids indexed on PubMed will have reached 26,227, with the number of publications increasing rapidly, making organoid research a burgeoning field of study, particularly in the area of single-cell transcriptomic sequencing (63) integrated with organoid research. In colorectal cancer research, the use of organoids has greatly advanced our understanding of the pathogenic mechanisms underlying this disease and has become a widely used tool in exploratory studies of its onset and progression (64) (**Figure 2A**). For example, Fengjiao Li and colleagues developed CRC organoids and subsequently extracted RNA for transcriptomic analysis, which revealed a significant correlation between the elevated expression of the ITGB7 and ITGA2B genes and sodium butyrate-induced apoptosis in these organoids. Additionally, sodium butyrate may induce cell cycle arrest and subsequent apoptosis by activating the PI3K−Akt signaling pathway (65). The utilization of organoids is also applicable to disease modeling and simulation. For example, Hao Zheng and colleagues established a colorectal cancer organoid biobank and demonstrated the induction of drug resistance following repeated exposure to low-dose chemotherapeutic agents. On this basis, the team developed a specific monoclonal antibody (LGR4-mAb) that can precisely block the LGR4-Wnt signaling pathway. Further studies demonstrated that LGR4-mAb not only effectively inhibited LGR4-Wnt signaling but also significantly enhanced drug-induced iron-induced apoptosis. The combination of LGR4-mAb with chemotherapeutic agents targeting the Wnt signaling pathway resulted in a notable increase in iron apoptosis in the organoids. This study illustrates the potential benefits of antibodies in CRC therapy. However, further studies are needed to analyze the heterogeneity of organoid responses to chemotherapeutic agents in greater depth (66). The use of organoids enables the discovery and validation of new therapeutic targets by accurately mimicking the microenvironment of human colorectal cancer *in vitro*. For example, Leon P. Loevenich’s research team discovered that NLE1 is capable of limiting the biosynthesis of ab initio proteins and the tumorigenic potential of advanced colorectal cancer cells (67). This significant outcome not only demonstrates the potential therapeutic efficacy of NLE1 but also provides a more precise targeting strategy to enhance the treatment of metastatic colorectal cancer in comparison with conventional approaches.

In conclusion, in the investigation of molecular mechanisms and prospective therapeutic targets, the use of gene editing and modification techniques in organoids has been explored. It has been employed in several fields, including tumor mechanism research, genomics, and the construction of tumor animal models. Nevertheless, there are still some limitations to the use of colorectal cancer organoids in basic research. These include the nonspecificity of sample collection, the impact of time-point differences on study accuracy, the diversity of cohorts that may lead to an underestimation of the relevance of treatment response, the problems posed by the limited number of human-derived colorectal cancer organoids and small sample sizes, and the validity of the drug screening methodology, which needs to be further validated (68).

### Drug research and development: the application of organoids in CRC

A further crucial application of organoid models is their capacity for high-throughput (69) drug screening *in vitro (* 68). The introduction of specific compounds into organoids to simulate the influence of drugs on cellular proliferation, differentiation, and function allows the assessment of potential adverse effects of treatment (70, 71) (**Figure 2A**). The standardization and large-scale production of organoid technology still present significant challenges, which may impact its potential for a wide range of clinical applications. Multicenter clinical studies should be conducted to validate the efficacy of organoid drug screening and investigate its integration with immunological and targeted therapies, among other approaches, to achieve a more comprehensive cancer therapeutic effect (9, 72). Concurrently, a drug screening program utilizing organoids has been initiated. These organoids serve as a highly realistic platform for investigating the effects of drugs on tumor cells under conditions that closely approximate human physiological settings (73). For example, Zhongguang Luo’s research team established a biobank comprising 33 patients and 37 patient-derived high-risk colorectal adenoma organoids (HRCA-PDOs). A high-throughput, high-content HRCA drug screen of 139 compounds was subsequently conducted. The drugs metformin, BMS754807, panobinostat, and AT9283 were screened and identified as potentially effective treatments, and all demonstrated generally consistent inhibitory effects on HRCA-PDO (10). A high-throughput screen was recently conducted by Iram Fatima and colleagues utilizing an annotated library of 1,600 FDA-approved drugs. During the screening process, albendazole modulated RNF20 expression and promoted the apoptosis of colorectal cancer cells. This occurs by delaying the G2/M phase and inhibiting the antiapoptotic transcription of the Bcl2 family of proteins (74).

Organoids enable rapid assessment of the efficacy of multiple drugs against tumors and predict patient response to specific treatments. Organoids facilitate the study of tumor drug resistance mechanisms and the development of novel therapeutic drugs. The high cost of culturing and screening organoids requires specialized techniques and equipment, which limits their wide application in clinical practice. Currently, there are potential issues with the reproducibility of organoid screening results. The development of more efficient and economical organoid culture and screening methods is necessary to reduce costs and expand the range of organoid applications. Moreover, larger-scale clinical trials are needed to validate the value of organoid applications in clinical practice.

### Predictive models: organoids for evaluating personalized treatment responses in CRC

In the conventional approach to oncology, the majority of patients typically undergo aggressive tumor resection following a cancer diagnosis (75). However, outcomes can vary significantly among individuals (76). As the understanding of the molecular heterogeneity of tumors and the pharmacogenomics of cancer therapies have advanced, the concept of tailored treatment strategies has become increasingly prominent in the field of therapeutic innovation (77, 78) (**Figure 2A**).

Nevertheless, organoid models provide an invaluable tool for predicting and assessing therapeutic outcomes, enabling researchers to elucidate the diverse sensitivities of cancer subtypes to a spectrum of interventions and to devise patient-specific treatment regimens (79). These promising approaches have the potential to not only increase treatment efficacy and minimize adverse effects but also significantly prolong patient survival. For example, S. N. Ooft and colleagues cultured colorectal organoids from biopsies and exposed them to eight different drugs, namely, vistusertib (an mTOR inhibitor) (80, 81), capivasertib (an AKT inhibitor) (82–84), and selumetinib (a MEK inhibitor) (85, 86). The objective of this study was to assess the antitumor activities of gefitinib (an EGFR inhibitor) (87, 88), palbociclib (a CDK4/6 inhibitor) (89, 90), axitinib (a VEGFR inhibitor) (91, 92), gedatolisib (a PI3K/mTOR inhibitor) (93), and glasdegib (a SMO inhibitor) (94). Nineteen patients exhibited sensitivity to at least one of the drugs, with 16 responding to mTOR inhibitors, 5 to AKT inhibitors, 3 to MEK inhibitors, 5 to EGFR inhibitors, and 2 to PI3K/mTOR inhibitors. Furthermore, no patients demonstrated sensitivity to CDK4/6 inhibitors, VEGFR inhibitors, or SMO inhibitors (95).

As organoid culture technology continues to be optimized, its potential for application as a preclinical predictive model has increased. Nevertheless, the limited drug screening activity and nonuniversal predictive ability of organoids, in addition to lower *in vitro* growth thresholds, may result in the persistence of tumor growth. It is essential to determine whether *in vitro* organoid sensitivity can be used to predict *in vivo* clinical response, as well as to improve culture success and the clinical efficacy of treatments. In light of the accelerated disease progression observed in patients, it is imperative to devote greater attention to the design of drug screening programs (96–98).

### Regenerative medicine: novel tissue engineering materials and functional cell sources in CRC

Despite the considerable promise of organoid technology for modeling and regenerating human organs, significant challenges remain before it can be applied in a clinical setting. In particular, the majority of organoid culture systems continue to utilize animal-derived materials, such as matrix gels (99, 100), which limits the extensive range of potential applications within the human body and presents a significant challenge for clinical applications. Currently, the field of biomaterials is actively promoting research and development activities aimed at creating new materials with practical applications. Hyaluronic acid-gelatin hydrogels exhibit distinctive biocompatibilities (101). This represents a significant area of current research interest. For example, Xiaobei Luo’s research team encapsulated CRC PDOs in a three-dimensional hyaluronic acid-gelatin hydrogel and subsequently cocultured them with cancer-associated fibroblasts (CAFs). The hydrogels were found to maintain the key molecular features of the original patient tumors in the CRC PDO, as evidenced by RNA and whole exome sequencing. The initial findings indicated that the hydrogels were not conducive to the cultivation of CAFs. Nevertheless, a subsequent coculture strategy was developed to maintain the viability of both CRC PDOs and CAFs (102). The feasibility of extracting essential biochemical and mechanical characteristics without relying on biochemically undefined and mechanically invariant animal-derived matrices has been demonstrated. These findings may facilitate the development of frozen matrices with tissue-specific properties for the culture of other types of patient-derived tumor-like organs. Organoids are increasingly recognized as promising transplantation media and as a source of functional cells within the field of regenerative medicine for cellular therapies (**Figure 2B**) (103).

The feasibility of conducting proof-of-concept studies in animal models has been demonstrated by experimental evidence (104). A methodology for the generation of human organoid tissues with codeveloping resident immune cells has been developed, which can be employed to simulate inflammatory diseases and organize the developmental roles of resident immune populations. For example, Jorge O. Mu employed pluripotent stem cell cultures of human colonic organoids (HCOs) to successfully differentiate functional macrophages. The transcriptional properties of HCO macrophages are analogous to those observed in resident macrophages within human fetal gut tissue. Macrophages regulate cytokine secretion, respond to pro- and anti-inflammatory signals, and effectively phagocytose pathogenic bacteria. Following transplantation into mice, HCO macrophages remain stable in colonic organoid tissues and are firmly attached to epithelial cells, with no replacement by host macrophages (105). This approach demonstrated the capacity of HCO to generate pluripotent hematopoietic progenitors and functional tissue-resident macrophages and was also employed to evaluate the potential of the cells.

Organoids represent a significant advancement in regenerative medicine. Its high-throughput screening capability has led to the establishment of an efficient translational research platform for regenerative medicine. Research and application of organoids have the potential to facilitate tissue damage repair and organ regeneration. However, organoids face several limitations, including the inefficiency of transplantation and reliance on cancer models, transplantation site limitations, and cellular heterogeneity. It is important to standardize protocols for organoid transplantation to improve transplantation efficiency and experimental reproducibility; broaden the sources of organoids, including other tissues, organs and human-derived organoids, to meet diverse research needs; and establish a comprehensive system of functional assessments of organoids, including intestinal barriers and immune functions, to ensure their functionality (106).

## Challenges and limitations: organoids in colorectal cancer research

At present, organoid models are confronted with many challenges and constraints. On the one hand, employing costly growth factors and animal-derived matrix extracts increases the financial burden associated with the cultivation process. Conversely, the clinical trial process necessitates the utilization of specialized equipment, consumables, and specific technical operations, which results in a markedly elevated cost in comparison to traditional 2D cell culture (**Table 1**). For example, Matrigel, a widely used product produced by BD Biosciences (107), is more expensive and holds a leading position in industry. Although colorectal cancer organoids can be constructed relatively quickly, scaling them up to a scale suitable for high-throughput drug screening may require an additional 2–4 weeks and resource investment compared with that of cell lines (108).

**Table 1:** Comparison of colorectal cancer organoids and two-dimensional cell lines.

Tumor models	Construction cost	Time consumption for modeling	Degree of construction difficulty	Mimicking the tumor microenvironment	Maintenance times
organoids (46)	high	long	difficult	good	long
cell lines (114)	low	short	easy	general	short

Comprehensive analyses have demonstrated that organoids display remarkable potential in mimicking the tumor microenvironment and predicting drug responses. However, their high cost, lengthy culture cycle, and intricate technical requirements have somewhat constrained their popularity and application in several research fields. Despite the limitations of cell lines in the aforementioned areas, their simplicity of operation and reduced cost have made them pervasive tools in research. The challenge for researchers is effectively weighing the advantages and disadvantages of the use of organoids and cell lines in future research.

A further technical challenge for organoids is the development of a standardized and reproducible procedure that encompasses the maintenance, culture, cryopreservation, and processing of organoids. Because of the significant interpatient heterogeneity of organoids, it is important to optimize cell culture media to ensure cell survival and proliferation. In the development of culture protocols, it is essential to consider the impact of human factors, including operational errors and differences between laboratory equipment, which may have a significant impact on the reliability of experimental results. Furthermore, in high-throughput drug screening, a standardized seeding step is essential to ensure that the size of the organoid is relevant to its clinical context (109). Organoids lack the complexity of living organisms, which limits their use in multiple therapeutic assays (110). Additionally, heterogeneity in terms of tumor phenotypes, genotypes, and cellular composition is a crucial parameter (111). For example, Ning Li et al. revealed the advantages of the PDO in probing the functional interactions between colorectal cancer and the tumor microenvironment by mapping the cellular distribution of CRC patient samples and establishing a CRC tumor model in mice. Furthermore, a coculture model of CRC patient-derived organoids was employed (45), suggesting that PDOs can be utilized to explore the functional interactions between CRC and the tumor microenvironment.

The utilization of automated platforms has become increasingly prevalent in comparison with traditional organoid cultures, reducing the requisite time and human resources. However, the application of this technology is accompanied by several challenges, including inconsistent volume measurements due to differences in solution viscosity, sample contamination, and possible damage to the sample caused by improper pipetting, which can lead to the accumulation of data errors and reduced reproducibility of experiments (112). For example, Diana Pinho and colleagues developed a novel low-cost microfluidic device, the organoid chip, for the culture and amplification of colorectal cancer organoids. Compared with the traditional 24-well plate culture method, the organoid chip not only markedly increased the survival rate and proliferation activity of colorectal cancer organoids but also significantly improved the formation efficiency and overall size of the organoids (113).

In conclusion, the CRC organoid model offers a comprehensive understanding of tumor biology, tumor heterogeneity, and therapeutic response through the combination of coculture with multiple cell types and multiomics analyses (97). These findings are expected to increase the identification of novel drug targets and diagnostic biomarkers and provide support for in-depth probing of the mechanism of action of a drug or mechanisms of resistance, thereby facilitating more efficient clinical translation (39).

## Future applications prospects

The field of organoid technology has undergone considerable advancements and evolution over the past few decades. This has involved overcoming numerous technical constraints, more rigorously assessing the fidelity of colorectal physiology, and demonstrating the practical utility of this technology. Researchers have sought to increase the fidelity of organoid physiology by optimizing culture formats and conditions (115). For example, organoid microarray technology (116) has been developed for high-throughput drug screening, whereas microfluidics has been employed for coculture (117). Inconsistencies in organoid characteristics have been observed across different culture protocols, emphasizing the urgent need to establish uniform criteria to define the characteristics of a true organoid (118).

The advent of new technologies has led to overcoming some of the earlier technical barriers, including those related to technical complexity and the necessity for experimental standardization (119). The experimental methods have now reached a point of maturity and are beginning to be translated into clinical applications. These developments are anticipated to facilitate a multitude of applications across a range of fields, including angiogenesis and coculture. For example, key domains such as organoid microarray technology, personalized precision medicine, drug screening and evaluation, biospecimen library construction, drug toxicity assessment, gene and cell therapies, regenerative medicine, and organ transplantation will undoubtedly benefit from these technological advancements (**Table 2**) (119, 120). As organoid technology continues to evolve, its applications in colorectal cancer research are anticipated to not only become more diverse but also significantly enhance our comprehension of the disease and provide invaluable assistance in improving patient survival rates (121).

**Table 2:** The primary benefits of organoids in various applications.

Features	Patient-derived xenografts	Cell lines	Organoids
biological stability	++	+	++
ease of downstream assays	+	+++	+++
high-throughput drug screening	–	+++	+++
low-throughput drug screening	+	+++	+++
genetic manipulation	–	+++	+++
cost benefits	–	+++	++
3D growth	+++	+/-	+++
cancer subtype modeling	–	+	+++
heterogeneity	++	–	+
ease of maintenance	–	+++	++
personalized treatment	–	–	+++
success rate of initiation	++	+	+++
time consumption for modeling	+++	+	+

This table is summarized in [ (24–44)]. +++, Best; ++, suitable; +, possible; –, unsuitable.

Organoids are more suitable for applications in personalized medicine than cell lines and patient-derived xenograft models, such as success rates of initiation, personalized treatment, cancer subtype modeling, complexity of disease models, 3D growth, genetic manipulation, low-throughput drug screening, and high-throughput drug screening.
